# Experience with Quality Assurance in Two Store-and-Forward Telemedicine Networks

**DOI:** 10.3389/fpubh.2015.00261

**Published:** 2015-11-26

**Authors:** Richard Wootton, Joanne Liu, Laurent Bonnardot, Raghu Venugopal, Amanda Oakley

**Affiliations:** ^1^Norwegian Centre for Integrated Care and Telemedicine, University Hospital of North Norway, Tromsø, Norway; ^2^Faculty of Health Sciences, University of Tromsø, Tromsø, Norway; ^3^Médecins Sans Frontières International, Geneva, Switzerland; ^4^EA 4569 Department of Medical Ethics and Legal Medicine, Paris Descartes University, Paris, France; ^5^Fondation Médecins Sans Frontières, Paris, France; ^6^GS-480 RFE, Toronto General Hospital, Toronto, Ontario, Canada; ^7^Department of Dermatology, Waikato Hospital, Hamilton, New Zealand; ^8^Waikato Clinical Campus, University of Auckland, Hamilton, New Zealand

**Keywords:** telemedicine, telehealth, quality assurance, quality control, LMICs

## Abstract

Despite the increasing use of telemedicine around the world, little has been done to incorporate quality assurance (QA) into these operations. The purpose of the present study was to examine the feasibility of QA in store-and-forward teleconsulting using a previously published framework. During a 2-year study period, we examined the feasibility of using QA tools in two mature telemedicine networks [Médecins Sans Frontières (MSF) and New Zealand Teledermatology (NZT)]. The tools included performance reporting to assess trends, automated follow-up of patients to obtain outcomes data, automated surveying of referrers to obtain user feedback, and retrospective assessment of randomly selected cases to assess quality. In addition, the senior case coordinators in each network were responsible for identifying potential adverse events from email reports received from users. During the study period, there were 149 responses to the patient follow-up questions relating to the 1241 MSF cases (i.e., 12% of cases), and there were 271 responses to the follow-up questions relating to the 639 NZT cases (i.e., 42% of cases). The collection of user feedback reports was combined with the collection of patient follow-up data, thus producing the same response rates. The outcomes data suggested that the telemedicine advice proved useful for the referring doctor in the majority of cases and was likely to benefit the patient. The user feedback was overwhelmingly positive, over 90% of referrers in the two networks finding the advice received to be of educational benefit. The feedback also suggested that the teleconsultation had provided cost savings in about 20% of cases, either to the patient/family, or to the hospital/clinic treating the patient. Various problems were detected by regular monitoring, and certain adverse events were identified from email reports by the users. A single aberrant quality reading was detected by using a process control chart. The present study demonstrates that a QA program is feasible in store-and-forward telemedicine, and shows that it was useful in two different networks, because certain problems were detected (and then solved) that would not have been identified until much later. It seems likely that QA could be used much more widely in telemedicine generally to benefit patient care.

## Introduction

Telemedicine can be broadly defined as any healthcare activity carried out at a distance. One common application of telemedicine concerns long-distance clinical consultation, where the doctor being consulted may be located in a different city or country from the patient. This reduces travel time and costs, and improves access to specialist healthcare. The use of telemedicine has grown in recent years as equipment has become more sophisticated, telecommunication networks have become more widespread and reliable, and the need for efficient use of healthcare resources has become more pressing. The assumption underlying the present study is that in any healthcare activity that has become routine procedure (i.e., it is not research), some sort of quality assurance (QA) will be desirable, perhaps mandatory. QA is the systematic process of checking regularly to see whether a product or service continues to meet specified requirements (1). Such a process will increase both customer confidence and the provider’s credibility, and will improve the efficiency of the work being carried out. The important features of a QA program have been summarized as (1)

1. a focus on the organization’s mission and the customer’s needs
2. a systematic approach to improvement, e.g., using the plan-do-check-act cycle, which offers a scientific method for continuous process improvement
3. stimulation for the development of human resources
4. facilitation of long-term thinking
5. the commitment of every participant.

Thus, a QA program will enable the people responsible for the service to satisfy themselves that its quality is being maintained. It will also allow them to identify problems at an early stage, with a view to solving them.

Quality assurance has been little used in telemedicine, apart from in image-based areas, such as radiology (2), retinal screening (3), or cytology (4). Liddy et al. added routine collection of user feedback data in a store-and-forward telemedicine network – this might be regarded as informal QA (5). Some guidance on routine audit of teledermatology services has also been published (6). Although QA is uncommon in telemedicine at present, there is no reason in principle why it cannot be applied more widely. In particular, QA of consultations taking place by telemedicine is an area of interest, whether such consultations occur in real time (e.g., by video) or asynchronously (e.g., by email).

The Collegium Telemedicus system is a general purpose system for managing telemedicine consultations (7). It is aimed at organizations which deliver services in low-resource settings, although its application is wider than that. Organizations which have successfully established telemedicine networks using the Collegium system may wish to conduct a program of QA; the Collegium system, therefore, includes a number of tools for this purpose. To date, there has been no published evidence relating to their use.

### QA in Telemedicine

We have previously proposed a framework for assessing telemedicine networks which provide teleconsultation services to doctors (8). The framework includes performance indicators for each of the three user groups (i.e., referrers, experts, and case coordinators). In addition, the framework covers the societal perspective, based on information about clinical- and cost effectiveness and integration with the conventional health care system, see Table 1.

**Table 1:** **Performance measurement framework for assessing telemedicine networks that provide teleconsultation services to doctors (8)**.

Performance indicator	Measurement possible in the CT system?
**Requester’s perspective**	
1. Rate of query arrival (new cases)	Directly
2. Proportion of failed queries	Indirectly (from web interface)
3. Time to first reply from an expert	Directly
4. Quality of replies	Directly
5. Ease of system usage	Indirectly (from user feedback)
**Case coordinator’s perspective**	
1. Rate of query arrival	Directly
2. Time required	Not measured
3. Resources available	Indirectly (from web interface)
4. Feedback from experts/feedback on patient outcomes	Directly
5. Ease of system usage	Not measured
**Expert’s perspective**	
1. Rate of requests received (for those experts who received queries)	Directly
2. Time to answer	Directly
3. Relevance to own expertise	Not measured
4. Feedback on patient outcomes	Directly
5. Ease of system usage	Indirectly (from user feedback)
**Societal perspective**	
1. Clinical effectiveness	Indirectly (from outcomes data)
2. Cost effectiveness	Indirectly (from outcomes data and user feedback)
3. Integration into the health care system, e.g., involvement of local people	Not measured

This framework represents a basic method of performance ­measurement with which teleconsultation networks can be measured and compared with one and other. However, QA requires long-term monitoring, rather than intermittent snapshots. Thus, a QA program depends on being able to measure appropriate performance indices but also requires methods for interpreting the resulting time-series data. In the Collegium system, the tools for QA include

1. performance reporting (on demand and automated) to assess trends
2. automated follow-up of patients to obtain outcomes data
3. automated surveying of referrers to obtain user feedback
4. retrospective assessment of randomly selected cases to assess quality.

These tools allow the majority of the indicators in the framework to be monitored, either directly or indirectly, see Table 1. The interpretation itself can be done in two ways. Graphs of performance statistics are useful for detecting long-term trends, but a more powerful method of obtaining early warning of adverse changes is by use of control charts that allow the sources of variation to be identified (9). In particular, common-cause variation (the usual, random variation which occurs in any process) can be differentiated from special-cause variation (which may indicate a change in the process requiring further investigation).

### Aim

The aim of the present study was to investigate the feasibility of using these tools in two mature telemedicine networks, and to review the results obtained. The hypothesis was that appropriate QA tools can be usefully applied to telemedicine networks, producing results that will assist clinical care and effective management.

## Materials and Methods

### Study Design

The feasibility of QA was examined in two Collegium networks over a period of 2 years, from August 2013 to July 2015 inclusive. The experience was reviewed by two telemedicine researchers at the end of the study period. Ethics permission was not required, because patient consent to access the data had been obtained and the work was a retrospective chart review conducted by the staff of the organizations concerned in accordance with their research policies (10).

### Networks

The Médecins Sans Frontières (MSF) network provides ­teleconsultations across a wide range of medical, surgical, and allied health disciplines, for health care workers in low-resource settings (11). During the study period, 122 referrers consulted 200 specialists about a total of 1241 cases. The New Zealand Teledermatology (NZT) network provides teledermatology consultations for general practitioners in certain health regions of New Zealand (12). During the study period, 57 referrers consulted four dermatologists about a total of 639 cases. The two networks, therefore, differed in size, workload, client base, and setting, thus providing contrasting conditions for the study.

### QA Program

The QA program implemented in the two networks was based on regular monitoring of

1. performance indicators, which were made available to case coordinators on demand via the system’s web interface. The indicators included the case referral rate (all cases, and in selected specialties), the time to allocation to a specialist, and the time to first response. Each month, summary reports of selected performance indicators were also sent automatically to the case coordinators by email
2. outcomes data. Information on patient outcomes was solicited from all referrers automatically. The system requested completion of a short progress report containing questions about the patient (13).
3. user feedback. Information about the management of the case, any benefits from the consultation, and the referrer’s opinion of the service generally was solicited from all referrers automatically. The system requested completion of a short progress report containing user-feedback questions (13).
4. overall teleconsultation quality, which was assessed each month by a QA review panel. We have previously described the measurement of teleconsultation quality, i.e., the “output” from the network (14). A panel of appropriately qualified observers responds to a questionnaire relating to a randomly chosen past case. The review panels were open to all network coordinators. The answers to the questionnaire allow two different dimensions of quality to be assessed: the quality of the process itself and the outcome, defined as the value of the response to three of the four parties concerned, i.e., the patient, the referring doctor and the organization. It is not practicable to estimate the value to society by this technique.

In addition, the senior case-coordinators in each network were responsible for identifying potential adverse events from email reports received from users.

There were four case coordinators on the NZT network and 19 coordinators on the MSF network. Not all coordinators were active for the whole study period. All coordinators participated in some monitoring activities (e.g., receiving email reports and notifications of completed user feedback surveys) and one senior coordinator in each network participated in all monitoring activities and in adverse incident detection.

## Results

### Regular Monitoring

**Figure 1:**
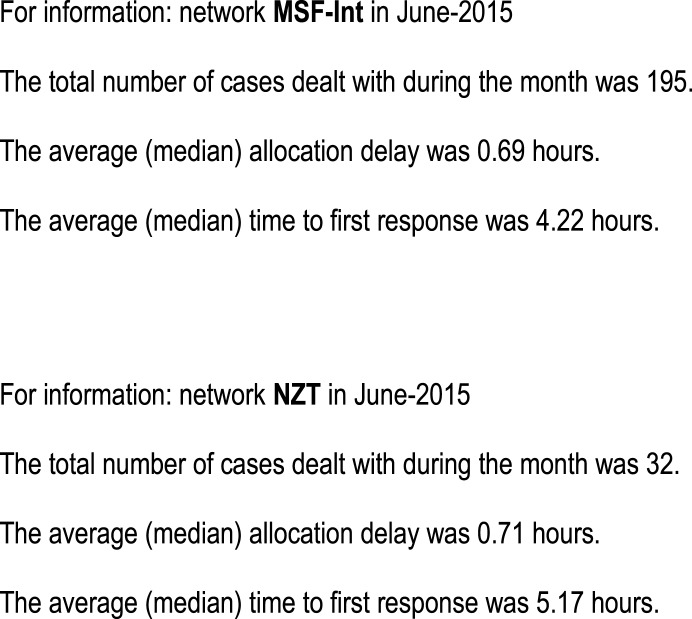
**Monthly email reports to the coordinators on the two networks**.

**Figure 2:**
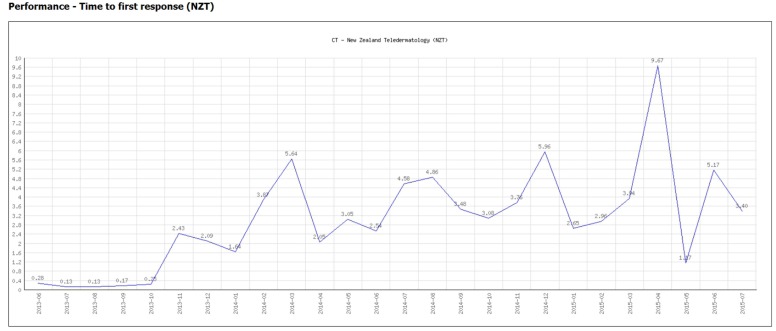
**Performance graph (produced on demand)**.

**Figure 3:**
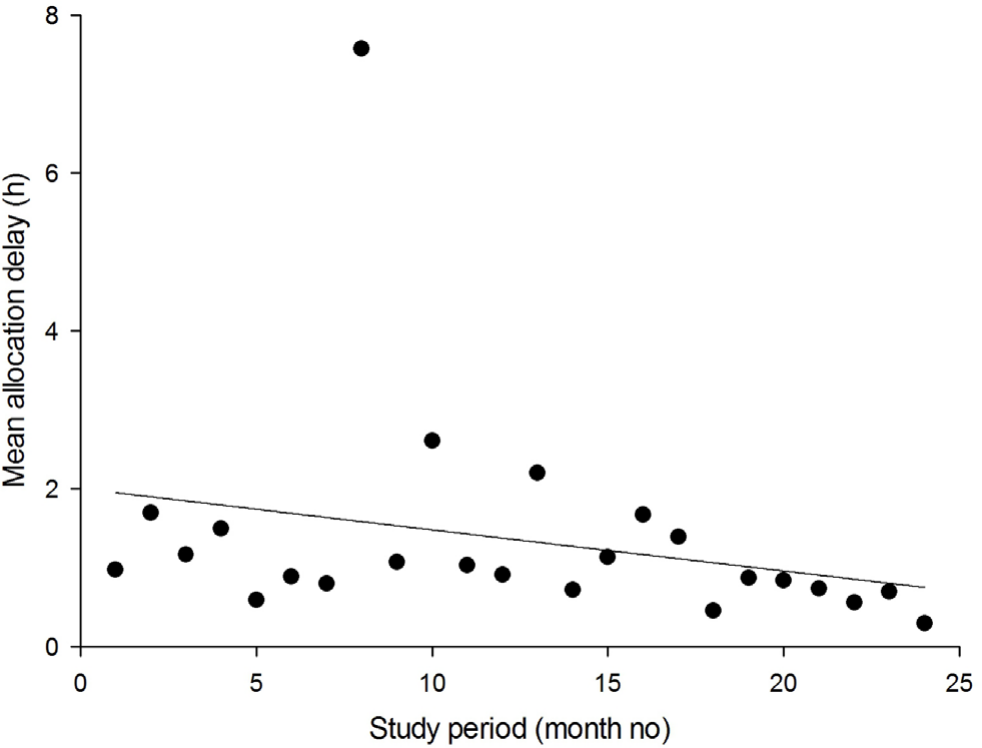
**Trend in the average delay in allocation of new cases (MSF network)**. The solid line is the linear regression. In month 8, there were 23 referrals. The apparent outlier in allocation delay at month 8 was caused by a single case in which an image dataset had to be sent by post, because the Internet connection at the field site in question did not allow a very large file to be uploaded.

**Figure 4:**
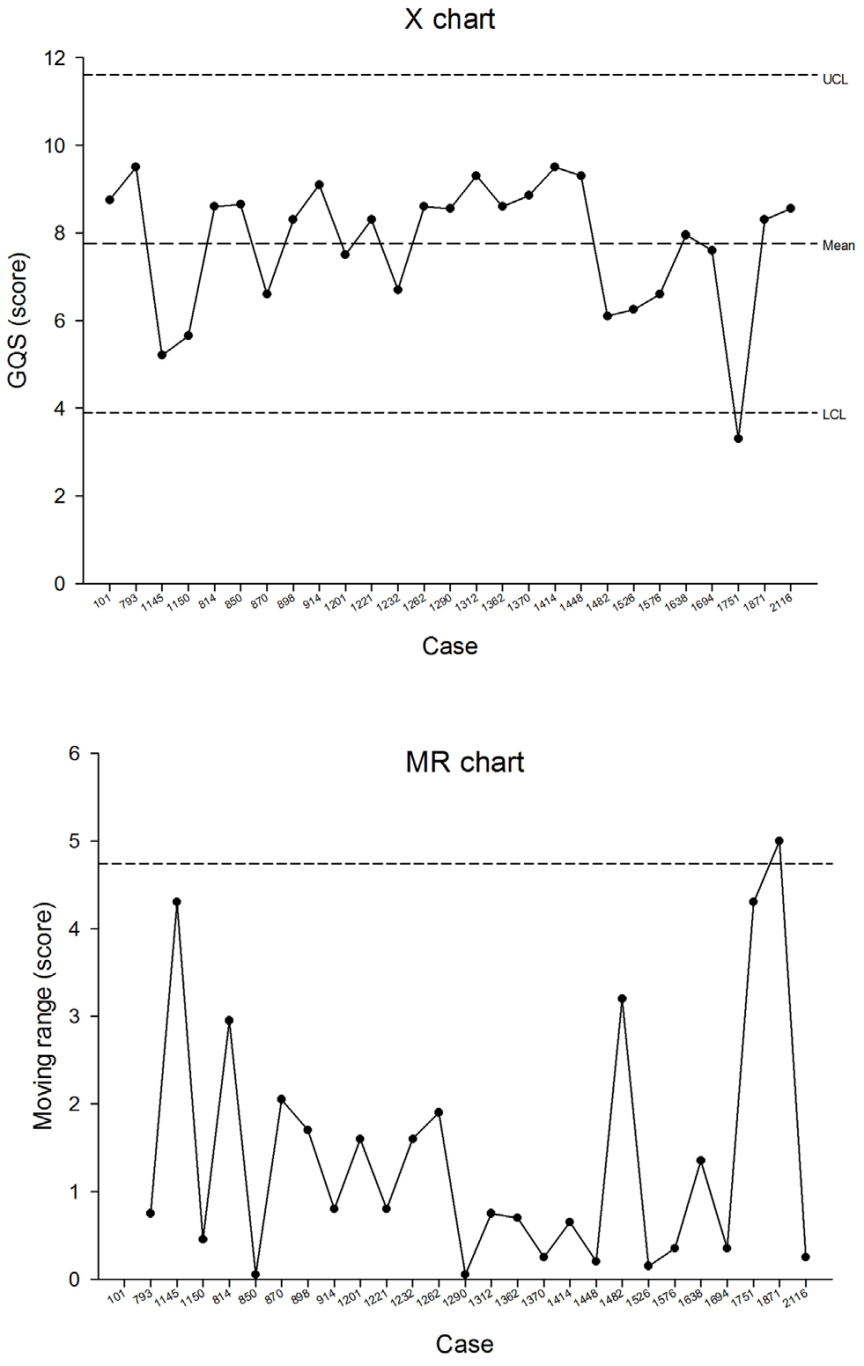
**Process control chart for the Grand Quality Score in the MSF network**.

1. Performance indicators. Monthly summary reports were sent to the coordinators, see Figure 1. In addition, plots were produced on demand, see Figure 2. These allowed the detection of trends in important network performance indices. For example, there was a trend to reduced allocation delay in the MSF network during the study (Figure 3). Although gratifying, the trend was not significant (*P* = 0.22).
2. Outcomes data. During the study period, there were 149 responses to the follow-up questions relating to the 1241 MSF cases (i.e., 12% of cases). During the study period, there were 271 responses to the follow-up questions relating to the 639 NZT cases (i.e., 42% of cases). The responses are summarized in Appendix 1. Referrers provided useful follow-up information on the cases, such as “Patient had a suspicious finding in the chest X ray which was inconsistent with the clinical course. But this was well clarified by the radiology expert.” (MSF) and “Mother says treatment cream is magic and is very happy” (NZT).
3. User feedback. The collection of user feedback reports was combined with the collection of patient follow-up data, thus producing the same response rates. The responses are summarized in Appendix 2. Referrers made very positive comments about the telemedicine service, such as “It is great to be able to have expert advice in a very short time. It helps a lot to evaluate better and to make the right decisions for unknown diseases/symptoms” (MSF) and “I think it is an excellent service. It helps to educate us GPs, if we can get advice on what a lesion is, then next time we see it we’ll know how to manage it better and therefore make fewer referrals to dermatologists” (NZT).
4. Overall teleconsultation quality. Monthly case reviews were carried out in both networks. One network used a single observer, and the second used a panel of observers. In the MSF network, a total of 123 assessments were carried out by the review panel and there were 222 non-responses (see Table 2). The process control chart for the Grand Quality Score indicated that there was a problem near the end of the study period, where special-cause variation was detected (MSF case 1751 fell outside the control limits), see Figure 4. This was investigated and remedial action was taken, see Table 3.

**Table 2:** **Number of assessments conducted by the QA review panel in the MSF network**.

Reviewer ID	No of assessments
1291	19
1332	19
2303	25
2305	7
2306	6
2310	12
2311	8
2312	18
2457	9

**Table 3:** **Examples of problems detected during the QA program**.

Network	Problem	Detected by	Analysis	Action
MSF	Field doctor requested a pediatric cardiology opinion; the case was sent to a specialist. Two days later, the referrer said, “the family is expecting me to talk to them tomorrow about possibilities, assuming the child will be fit enough for transport or discharge. Any chance I might have an answer by then?”	Coordinator message	The coordinator failed to follow progress (or lack of) on the case. Although it had been allocated to a pediatric cardiologist, the coordinator failed to notice that there had been no response	When the field doctor asked for a reply, several coordinators became involved with trying to find a pediatric cardiologist to respond at short notice. This was successful, but it was wasteful of resources. Daily summary reports were introduced for case coordinators to reduce the chance of unanswered cases being overlooked
MSF	Field doctor complained that “3 times, we have received 2 different answers for 1 case”	User feedback report	New referrals in the MSF network are normally sent to a single specialist only. If that specialist has not replied within 24 h, the query will then be sent to a second specialist. It was unfortunate that in the three cases concerned, the first specialist did not respond promptly, so the query was sent to a second specialist. Both specialists then replied. In reviewing the responses, there did not appear to be substantial disagreement in the advice offered to the referring doctor, although there was some potential for confusion	The complaint was followed up carefully. The referrer was reminded that as the treating doctor, management of the patient remains his responsibility, and it is entirely up to him how to interpret the advice received
MSF	Case 1751 fell outside the control limits (see Figure 4)	Monthly quality review	Investigation revealed that two coordinators had simultaneously allocated the case initially (luckily to the same team). Although there was an initial response from the pediatricians, it then took over a week to obtain an opinion from an infectious diseases specialist. There was very limited engagement with the referrer, which prevented the teleconsultation reaching a conclusion. There was no feedback on what happened to the patient	Modifications were subsequently made to the system software to reduce the chance that a case could be allocated simultaneously by two different case coordinators
NZT	Two different general practitioners reported not receiving the email notifying them that the specialist had responded	User feedback report	Investigation revealed that email originating from certain IP addresses was being filtered at a high level	The IT departments concerned were contacted. An information message was added to outgoing emails to remind referrers to login and check for responses if they were unsure of the status of their case

#### Problems Detected

During the 2-year study period, a number of problems were detected by regular monitoring. Some examples from the two networks are provided in Table 3.

In addition, various adverse events were identified in each network. For example, in the MSF network, there were several telemedicine cases for which appropriate specialists were not available, and new specialists had to be recruited at short notice. In the NZT network, there were instances of duplicate case ­numbering (data subsequently amalgamated), misunderstandings about image uploading (help documentation updated), difficulties in using the system with a mobile device (mobile interface developed), missing images (systems error corrected by IT staff), large files not ­uploading (file transfer problem ­corrected), and difficulty in finding a specific case (free-text keyword search function added).

### Discussion

In the present study, the feasibility of conducting a comprehensive QA program was examined in two relatively stable and mature telemedicine networks. Thus, the nature of the study was operational research, rather than clinical research, and the methodology employed was able to take advantage of existing systems. We are not aware of previous similar work.

#### Outcomes Data

The limited outcomes data available for MSF cases suggested that the telemedicine advice proved useful for the referring doctor in the majority of cases and was likely to benefit the patient (Appendix 1). The percentage of requests for follow-up information that were fulfilled in the two networks was much lower in the MSF network, presumably because of the high turnover of field staff, and patients being discharged and, therefore, lost to follow up. Conversely, most referrers in the NZT network were stable general practitioners, who closely followed up their patients. There may also have been a response bias whereby referrers with positive experiences tended to respond to requests for follow-up information, whereas those with negative experiences did not.

#### User Feedback

The user feedback was overwhelmingly positive (Appendix 2). It also suggested that the teleconsultation had provided cost savings in about 20% of cases, either to the patient or family, or to the hospital/clinic treating the patient. The comments provided by the referrers suggested that the cost savings to the patient or family were mainly due to avoided journeys, i.e., that telemedicine removed the need for a trip to hospital for a specialist appointment; this was the case in both networks. The comments provided by the referrers suggested that the cost savings to the hospital or clinic were also mainly due to avoiding unnecessary referrals.

#### Comparison Between the Two Networks

Three important questions concerning patient follow-up and five important questions concerning user feedback were compared between the two networks. In all eight questions, the proportion of responders answering “yes” was higher in the NZT network than in the MSF network, Table 4. However, the differences were not significant in three of the questions. The largest differences were in the question about whether the eventual outcome would be beneficial for the patient (75% of NZT responders vs. 51% of MSF responders) and whether the referrer was able to follow the advice provided (94% of NZT referrers vs. 70% of MSF referrers). This probably reflects the different settings of the two networks. For example, the NZT network deals with a single specialty (dermatology), whereas the MSF network deals with a very wide range of medical, surgical, nursing, and allied health matters. In addition, MSF referrers operate in resource-constrained settings, which often limit their diagnostic and therapeutic options.

**Table 4:** **Summary responses – comparison between networks**.

Question	MSF		NZT		Difference in percentage answering “yes”	*P*-value*
	No. of definite responses	Percentage responding “yes”	No. of definite responses	Percentage responding “yes”		
**Patient follow-up**						
Q7-3. Advice found to be helpful – did it improve the patient’s symptoms?	106	42	174	53	11	0.08
Q7-4. Advice found to be helpful – did it improve function?	99	36	153	41	5	0.45
Q8. Do you think the eventual outcome will be beneficial for the patient?	132	51	256	75	24	<0.001
**User feedback**						
Q4. Were you able to follow the advice given?	147	70	267	94	24	<0.001
Q6. Did you find the advice helpful?	145	94	263	99	5	0.001
Q7-1. Advice found to be helpful – did it clarify your diagnosis?	116	82	242	91	9	0.01
Q7-2. Advice found to be helpful – did it assist with your management of the patient?	130	92	250	98	6	0.01
Q9. Was there any educational benefit to you in the reply?	147	93	260	94	1	0.60

*Based on the *Z*-test for comparing two independent proportions.

#### Control Charts

Over a 2-year study period, one aberrant quality reading was detected. Further investigation found that in providing a response to the referrer, various problems had occurred that reduced the quality of the resulting consultation (see Table 3).

#### Points Arising

There could be better integration of the outcomes data with the performance indices. Further software development is required in order to allow ready visualization of outcomes data.

#### Strengths and Weaknesses

The present study was conducted in two operational telemedicine networks, and was therefore, based on real, not simulated data – this represents a strength. The results represent a validation of the service being delivered, demonstrating its value to the patients, users, and organizations involved. Audit and quality improvement of this kind are aligned with contemporary thinking about the importance of overseeing service function and safety. These are all strengths.

However, the judgment about the value of the QA activities as a whole was subjective. That is, it was not feasible to conduct a study in which a telemedicine network using QA was compared with a control telemedicine network that did not use QA. In conducting the QA program, several problems came to light, and were dealt with, which might otherwise have gone unnoticed. But it is hard to *prove* this. What we can say is that the MSF network had operated for 4 years before the QA program was introduced, and in this period, there was obviously no formal monitoring of performance data. Thus, the QA process enforced a discipline of monitoring, leading to the early detection of problems.

#### Future Work

The present study shows that QA is feasible in the two networks studied, and also suggests that it is useful. The next stage must be the organizational adoption of a comprehensive QA program for telemedicine by the organizations concerned. Such a program would include procedures to review reports, monitor cases/users and check data, as described in the present paper. In addition, a comprehensive QA program would investigate protocol compliance, to identify protocol violations. For example, anecdotal reports from several of the Collegium networks suggests that problems are often caused unintentionally by users who upload files in proprietary formats (despite automatically generated warnings in the software, and the provision of user documentation explaining that the intended recipient of a file in a non-open source format may be unable to read it). There have also been problems caused by referrers who submit the same case more than once. Finally, a comprehensive QA program would include the measurement of service availability, e.g., web site performance.

### Conclusion

The present study demonstrates that a QA program is *feasible* in store-and-forward telemedicine. Experience over the first 2 years in two different networks indicates that QA is also *useful*, because certain problems were detected (and then solved) that would not have been identified until much later. The QA tools available via the Collegium system worked well and provided the foundation for a comprehensive QA program. QA elements examined in the two different networks produced similar findings. Thus, the hypothesis that QA can be applied effectively in a teleconsulting network was supported. It seems likely that QA could be used much more widely in telemedicine generally.

### Conflict of Interest Statement

The authors declare that the research was conducted in the absence of any commercial or financial relationships that could be construed as a potential conflict of interest.

## Figures and Tables

**Figure S1:**
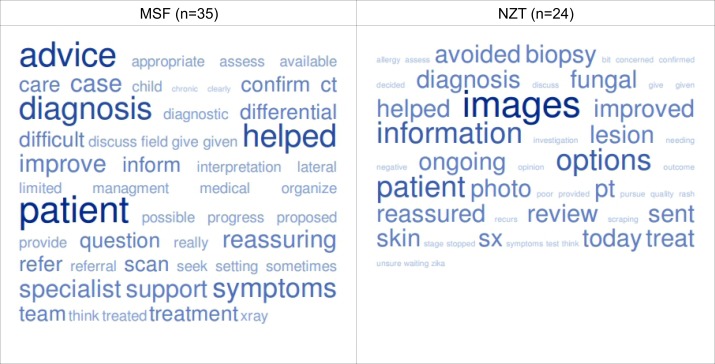
Word frequencies* in the free-text responses to the invitation to comment about why the advice was helpful in the case being followed up. *See http://tagcrowd.com/.

**Figure S2:**
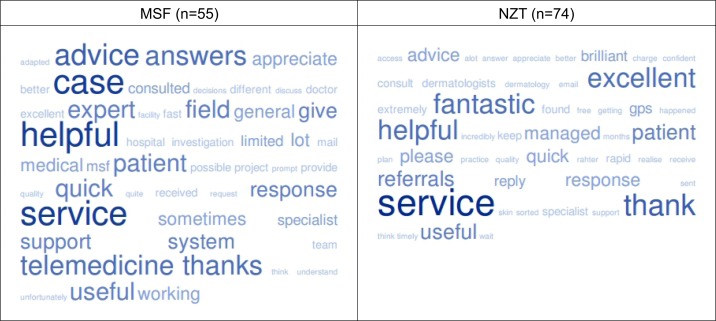
Word frequencies* in the free-text responses to the invitation to comment about the telemedicine service.*See http://tagcrowd.com/.

## Appendix 1

**Table:** **Summary of patient follow-up**.

	Yes	Perhaps	No	Skipped	Yes (% responses)	Positive (% responses)*
A. MSF network (*n* = 149)						
Q7-3. Advice found to be helpful – did it improve the patient’s symptoms?	45	N/A	61	43	42	42
Q7-4. Advice found to be helpful – did it improve function?	36	N/A	63	50	36	36
Q7-5. Advice found to be helpful – any other reason?	35 comments, see Figure S1			114	N/A	N/A
Q8. Do you think the eventual outcome will be beneficial for the patient?	67	47	18	17	51	86
B. NZT network (*n* = 271)						
Q7-3. Advice found to be helpful – did it improve the patient’s symptoms?	93	N/A	81	97	53	53
Q7-4. Advice found to be helpful – did it improve function?	63	N/A	90	118	41	41
Q7-5. Advice found to be helpful – any other reason?	24 comments, see Figure S1			247	N/A	N/A
Q8. Do you think the eventual outcome will be beneficial for the patient?	191	60	5	15	75	98

**i.e. those answering Yes or Perhaps*.

## Appendix 2

**Table:** **Summary of referrer feedback**.

	Yes	No	Don’t know	Skipped	Yes (% responses)
A. MSF network (*n* = 149)					
Q1. Was the case sent to an appropriate expert?	140	2	7	0	94
Q2. Was the answer provided sufficiently quickly?	134	14	1	0	90
Q3. Was the answer well-adapted for your local environment?	127	21	N/A	1	86
Q4. Were you able to follow the advice given?	103	44	N/A	2	70
Q5. If NO, could you explain briefly why not?	53 comments			96	N/A
Q6. Did you find the advice helpful?	136	9	4	0	91
Q7-1. Advice found to be helpful – did it clarify your diagnosis?	95	21	N/A	33	82
Q7-2. Advice found to be helpful – did it assist with your management of the patient?	119	11	N/A	19	92
Q9. Was there any educational benefit to you in the reply?	136	11	N/A	2	93
Q10-1. Was there any cost-saving for the patient/family as a result of this consultation?	29	79	32	9	21
Q10-2. If YES, please explain briefly	26 comments			123	
Q10-3. Was there any cost-saving for the hospital/clinic as a result of this consultation?	28	59	18	44	27
Q10-4. If YES, please explain briefly	27 comments			122	
Q11. Please add any other comments about this case specifically	56 comments			93	
Q12. Please add any other comments about the service generally	55 comments, see Figure S2			94	
B. NZT network (*n* = 271)					
Q1. Was the case sent to an appropriate expert?	269	1	1	0	99
Q2. Was the answer provided sufficiently quickly?	268	2	1	0	99
Q3. Was the answer well-adapted for your local environment?	268	2	N/A	1	99
Q4. Were you able to follow the advice given?	250	17	N/A	4	94
Q5. If NO, could you explain briefly why not?	22 comments			249	N/A
Q6. Did you find the advice helpful?	261	2	4	4	98
Q7-1. Advice found to be helpful – did it clarify your diagnosis?	220	22	N/A	29	91
Q7-2. Advice found to be helpful – did it assist with your management of the patient?	244	6	N/A	21	98
Q9. Was there any educational benefit to you in the reply?	244	16	N/A	11	94
Q10-1. Was there any cost-saving for the patient/family as a result of this consultation?	160	57	29	25	65
Q10-2. If YES, please explain briefly	125 comments			146	
Q10-3. Was there any cost-saving for the hospital/clinic as a result of this consultation?	132	45	25	69	65
Q10-4. If YES, please explain briefly		99 comments		172	
Q11. Please add any other comments about this case specifically	68 comments			203	
Q12. Please add any other comments about the service generally	74 comments, see Figure S2			197	
